# Characterizing Microstructural Evolution of TP304 Stainless Steel Using a Pulse-Echo Nonlinear Method

**DOI:** 10.3390/ma13061395

**Published:** 2020-03-19

**Authors:** Yichen Liu, Xiongbing Li, Guangdong Zhang, Shuzeng Zhang, Hyunjo Jeong

**Affiliations:** 1 School of Traffic and Transportation Engineering, Central South University, Changsha 410075, China; 15173929277@163.com (Y.L.); lixb_ex@163.com (X.L.); guangdongzhang1995@163.com (G.Z.); 2 Key Laboratory of Traffic Safety on Track, Ministry of Education, Central South University, Changsha 410075, China; 3 Department of Mechanical Engineering, Wonkwang University, Iksan 54538, Korea

**Keywords:** TP304 stainless steel, microstructural evolution, nonlinear acoustics, pulse-echo method, absolute nonlinear parameter measurement

## Abstract

Tube/Pipe (TP) 304 stainless steel has been widely used in industry, but a change in its microstructures may endanger its service safety, and it is essential to evaluate its microstructural evolution. In this work, a pulse-echo nonlinear method is proposed to characterize the microstructural evolution of the TP304 stainless steel. The detailed pulse-echo nonlinear experimental process is presented, and it is shown that the absolute nonlinear parameter can be determined when the effect of attenuation is taken into account. The microstructural evolution of TP304 stainless steel is artificially controlled by annealing treatments before it is evaluated by using nonlinear ultrasonic method and metallographic method. The results show that the grain sizes increase as the annealing time increases, which leads to the performance degradation of the TP304 steel and an increase in the nonlinear parameters, with the reason discussed considering the variation in the microstructure. The present pulse-echo nonlinear method is easier to conduct than the traditional transmission-through method and the absolute nonlinear parameter can be determined for quantitative characterization. The variation in determined nonlinear parameters provides a reference to evaluate the microstructural evolution of TP304 stainless steel.

## 1. Introduction

Tube/Pipe (TP) 304 stainless steel has numerous advantages including high corrosion resistance, good plasticity, high formability, and high temperature resistance, and has been widely used in ships, the nuclear industry, and pipelines of petrochemical plants [1,2,3]. When these stainless steel components are in service, their microstructures may change due to the long-term continuous load, high temperature and high pressure. The poor evolution of the microstructure will lead to the degradation of the mechanical properties, which will endanger the safety of service. Therefore, it is essential to evaluate its microstructural evolution in a timely and effective way to ensure the service safety of components.

The ultrasonic test method provides an effective nondestructive test because the ultrasonic signals are directly related to the properties of the materials [4,5]. However, the traditional linear ultrasound is usually used for macro-flaws. Recently, nonlinear ultrasonic techniques have been considered a potential tool for the assessment of the microstructural evolution and mechanical properties of materials, and it is shown that the nonlinear acoustic parameter is sensitive to the changes of grain sizes, dislocations, precipitates, and fatigue microcracks [6]. Therefore, nonlinear acoustic testing can be used to characterize the degradation of the microstructural properties of TP 304 stainless steel components.

When a monochromic ultrasonic wave is transmitted in a material, the waveform is distorted by the nonlinear elastic property of the material and harmonic waves are generated [7]. The nonlinear ultrasonic method often measures these harmonic waves to obtain the relative or absolute nonlinear parameter to evaluate the microstructural evolution of materials. The typical method uses longitudinal waves and is usually conducted in through-transmission mode, which requires access to both sides; however, this technique may be restrictive in a field measurement scenario where, for example, access is only possible to the outer surface of a pipeline [8]. The pulse-echo method, which enables single-side access to the test component using a nonlinear longitudinal wave, provides a useful tool for practical applications of nonlinear ultrasonic measurement.

Determining the absolute nonlinear parameter is important in order to quantitatively evaluate the microstructural evolution of materials [9]. Measuring nonlinear parameters for fluids using the pulse-echo method with a rigid boundary has been reported [10]. For solid materials or structures, the stress-free boundary will destructively alter the nonlinear wave generation process. Some researchers have shown that the ‘residual’ nonlinear wave reflected from stress-free boundaries can still be measured [8,11]. However, there are some difficulties in determining the absolute nonlinear parameters of TP 304 stainless steel using the pulse-echo method. First, the circuit connection mode is uncertain, and it is found that selection and connection sequence of electrical elements will affect the measurement results. Second, the receiver should be calibrated to measure the absolute wave amplitude, and corrections for diffraction and reflection coefficient have to be made for calculating the accurate nonlinear parameters. Third, since the TP304 stainless steel has a larger attenuation coefficient than most fluids, the attenuation will affect the nonlinear parameter measurement results, and its effects have to be taken into account [12].

In this work, we develop a pulse-echo nonlinear method to evaluate the microstructural evolution of the TP304 stainless steel. The above-mentioned inadequacies will be solved to determine the absolute nonlinear parameter of the TP304 stainless steel. The process of measurement using pulse-echo method will be described in detail, and the electrical impedance mismatch problem [13,14] is solved in this work. This method is introduced to determine nonlinear parameters of annealed specimens. The relationship between the nonlinear parameters and microstructural evolution of materials is developed, and the potential applications using the pulse-echo nonlinear method are discussed.

## 2. Theory

### 2.1. Nonlinear Wave Propagation and Generation Process

**Figure 1:**
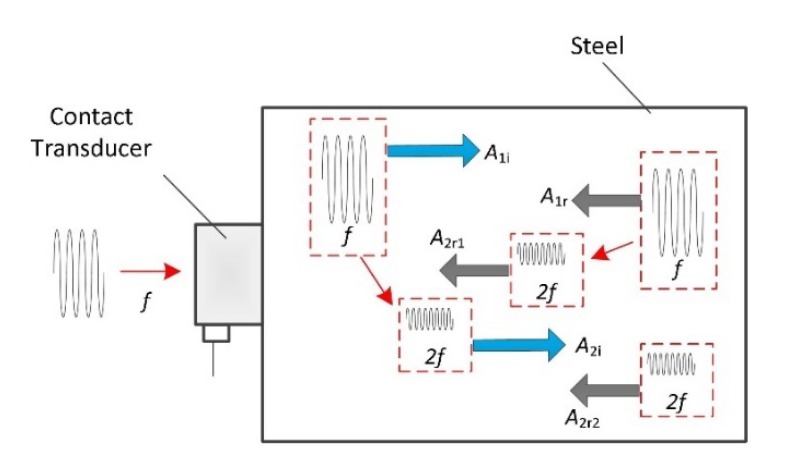
Schematic of a pulse-echo testing configuration with a stress-free boundary.

The nonlinear wave pulse-echo experiment process for characterizing the microstructural evolution of TP304 steel specimens is shown in Figure 1. One transducer works as both the transmitter and the receiver. In this condition, the waves generated by the transducer propagated in the specimen are reflected from the stress-free boundary and are then received by the same transducer. Due to the material properties, second harmonic waves (frequency of 2*f*) will be generated when the fundamental wave (frequency of *f*) propagates.

The nonlinear harmonic wave generation process can be described as follows. In the forward propagation direction, when the fundamental wave propagates, the second harmonic wave generated by the nonlinearity of steels will be generated. We use *A*_1*i*_ and *A*_2*i*_ to represent these fundamental and second harmonic wave displacements. In the backward propagation direction, *A*_1*r*_ represents the reflected wave when *A*_1*i*_ hits the boundary. As discussed in our previous study [14], the nonlinear wave after reflection consists of two components, *A*_2*r*1_ and *A*_2*r*2_, where *A*_2*r*1_ is the second harmonic generated by the reflected *A*_1*r*_ and *A*_2*r*2_ is the reflected second harmonic when *A*_2*i*_ reaches the boundary. The total second harmonic after reflection, *A*_2_, is thus obtained by adding *A*_2*r*1_ and *A*_2*r*2_.

By measuring the fundamental and second harmonic wave displacements, the nonlinear parameter, *β*, of the TP304 steel can be determined traditionally as [7]:

(1) $$\beta =\frac{8}{k^{2}z}\frac{A_{2}\left(z\right)}{A_{1}^{2}\left(z\right)}$$

where *k* is the wave number, and *z* is the propagation distance.

Note that Equation (1) is derived using the nonlinear plane wave. The nonlinear parameter determined using Equation (1) will be affected by the diffraction, attenuation and the reflection boundary when transducers with finite sizes are used [15]. Thus, the correction for these factors should be taken into account. In addition, if one wants to measure the absolute nonlinear parameter, the transducer should be calibrated.

### 2.2. Corrections for the Diffraction, Attenuation and Reflection

As shown in Figure 1, when a finite-size transducer is used to generate and receive nonlinear waves, the effects of the wave diffraction, attenuation and reflection coefficient should be included in the wave equation. Therefore, we introduce a nonlinear wave equation in a three-dimensional coordinate system to model the reflecting waves. The theoretical work has been done in our previous work [15]; here, we present the reflected wave distribution results modeled using multi-Gaussian beams, as:

(2) $$A_{1}\left(x,y,z\right)=R_{f}A_{0}\exp\left(ikz\right)\sum_{n=1}^{25}\frac{A_{n}\exp\left(-\alpha_{1}z\right)}{1-iB_{n}z/D_{R}}\exp\left(-\frac{B_{n}/a^{2}}{1-iB_{n}z/D_{R}}\left(x^{2}+y^{2}\right)\right)$$

(3) $$\begin{array}{c} A_{2}\left(x,y,z\right)=R_{f}\frac{\beta k^{2}A_{0}^{2}\exp\left(2ikz\right)}{4}\times \int_{0}^{z_{0}}\sum_{m=1}^{25}\sum_{n=1}^{25}\frac{-A_{m}A_{n}B_{b}\exp\left(-\alpha_{2}\left(z-z^{\prime }\right)-2\alpha_{1}z^{\prime }\right)}{\left(2z+B_{a}\right)z^{\prime }+B_{a}z-2B_{b}}\\ \times \exp\left\{-2ik\left(x^{2}+y^{2}\right)\left(\frac{\left({z^{\prime }}^{2}+B_{a}z^{\prime }-B_{b}\right)/\left(z-z^{\prime }\right)}{\left(2z+B_{a}\right)z^{\prime }+B_{a}z-2B_{b}}\right)\right\}dz^{\prime }\\ +R_{f}^{2}\frac{\beta k^{2}A_{0}^{2}\exp\left(2ikz\right)}{4}\times \int_{z_{0}}^{z}\sum_{m=1}^{25}\sum_{n=1}^{25}\frac{-A_{m}A_{n}B_{b}\exp\left(-\alpha_{2}\left(z-z^{\prime }\right)-2\alpha_{1}z^{\prime }\right)}{\left(2z+B_{a}\right)z^{\prime }+B_{a}z-2B_{b}}\\ \times \exp\left\{-2ik\left(x^{2}+y^{2}\right)\left(\frac{\left({z^{\prime }}^{2}+B_{a}z^{\prime }-B_{b}\right)/\left(z-z^{\prime }\right)}{\left(2z+B_{a}\right)z^{\prime }+B_{a}z-2B_{b}}\right)\right\}dz^{\prime } \end{array}$$

where *A_m_*, *B_m_*, *A_n_*, and *B_n_* are 25 groups of Gaussian coefficients [16], *B_a_* = *i*(*B_m_* + *B_n_*)/*D_R_*, and $B_{b}=B_{m}B_{n}/D_{R}^{2}$. *R_f_* is the reflection coefficient of the steel–air interface, *A*_0_ is the initial wave displacement, α is the radius of the transducer, *Z*_0_ is the thickness of the specimen, *Z* is the wave propagation distance, and α_1_ and α_2_ are the attenuation coefficients for the fundamental and second harmonic waves, respectively.

The average field received by the same transducer at *Z =* 2*Z*_0_ can be obtained by the following integral:

(4) $$\tilde{A_{n}}\left(z\right)=\frac{1}{S}\int_{S}A_{n}\left(x,y,z\right)dS$$

where *S* is the transducer area. Thus, the received average fundamental and second harmonic waves can be expressed more explicitly in terms of the plane waves and the corresponding corrections as:

(5) $${\tilde{A}}_{1}\left(z\right)=\left[A_{0}\exp\left(ikz\right)\right]\left[C_{T1}\left(z\right)\right]$$

(6) $${\tilde{A}}_{2}\left(z\right)=\left[\frac{\beta k^{2}A_{0}^{2}z}{8}\exp\left(ik2z\right)\right]\left[C_{T2}\left(z\right)\right]$$

where ${\tilde{C}}_{Tn}^{}(z)={\tilde{A}}_{n}(z)/A_{n}^{plane}(z),\;n=1,2$ represents the total correction for the received nth harmonic that incorporates the diffraction, attenuation and the boundary reflection.

Therefore, the nonlinear parameter measurement results using the plane wave solution, Equation (1), can be modified when these effects are included as:

(7) $$\beta =\frac{8}{k^{2}z}\frac{{\tilde{A}}_{2}\left(z\right)}{{\tilde{A}}_{1}{}^{2}\left(z\right)}\left[\frac{C_{T1}^{2}\left(z\right)}{C_{T2}\left(z\right)}\right]$$

## 3. Materials and Method

### 3.1. Specimen Preparation

Specimens of commercial TP304 stainless steel are prepared for experiments. The chemical composition of the investigated TP304 stainless steel is provided by a commercial company and shown in Table 1 for reference. Six specimens with dimensions 120 × 120 × 40 mm are prepared by cutting from one stainless steel plate. Annealing treatments are performed on these specimens to change microstructures and obtain different mechanical properties. Specimen (a) is not heat-treated. Specimens (b)–(f) are annealed at a constant temperature of 1080 °C for 2, 4, 6, 10, and 14 h, respectively.

Prior to nonlinear experiment testing, all the specimens are carefully polished using 400 to 2000 grit emery papers successively, so that the surface roughness and oxidation do not affect the ultrasonic measurement results.

In order to observe the variation in the crystal phases of these heat-treated specimens using a light microscope, the corrosive agent, HF:HNO_3_:H_2_O = 2:1:7, is configured and dropped onto the test piece and eroded for 20 min. Then, the hardness of each stage is determined using a Wolpert–Wilson micro-Vickers hardness tester.

### 3.2. Pulse-Echo Nonlinear Experiments

**Figure 2:**
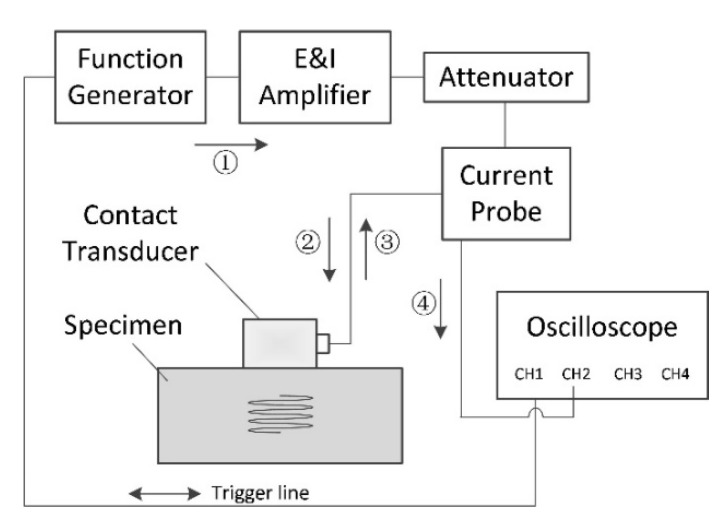
Experimental setup for pulse-echo nonlinear method. Serial numbers are used to denote the transfer order of signals.

The experimental setup for measuring nonlinear parameters using the pulse-echo method is shown in Figure 2. A waveform generator (33250A, Agilent Technologies, Inc., Santa Clara, CA, USA) is used to generate a 20-cycle toneburst signal with a center frequency of 3.5 MHz. The signal is linearly amplified by an amplifier (2100L, Electronics & Innovation, Ltd., Rochester, NY, USA), flows through a 6 dB attenuator and a current probe (Tektronix CT-2, Tektronix, Inc., Wilsonville, OR, USA), and drives a contact ultrasonic transducer, which is made of a LiNbO_3_ crystal with a diameter of 12.7 mm and a central frequency of 5 MHz. The transducer has a broad band, so that the fundamental wave at the frequency of 3.5 MHz and second harmonic wave at the frequency of 7 MHz can be well measured. The transducer is brought into contact with the specimen surface by a liquid coupling agent, and is fixed by applying a certain force using a clamping device. The wave propagates in the specimen, is reflected by the stress-free interface, and is received by the transducer. The received signal is digitized by an oscilloscope (LT332, LeCroy, Chestnut Ridge, NY, USA) and saved to the computer for data processing. The initial input voltage was 50 to 950 mV with an interval of 100 mV.

Note that the attenuator is an essential equipment to match the impedance difference for measuring the absolute nonlinear parameter in the pulse-echo nonlinear experiment. In addition, the attenuator should be located between the amplifier and current probe. Our experiments show that, when the attenuator is used, the resistance value for the input port is close to 50 Ω, and the proposed calibration method for the receiver can be directly used [13,14]. However, when there is no attenuator, the resistance value is very small, so that the system absorption for high frequency waves in the input port can be reduced and stable acoustic signals can be received by the transducer. The measurement results using the pulse-echo experimental setup will be discussed in Section 4.2.

In order to measure the absolute nonlinear parameter of the TP304 stainless steel specimens, the contact transducer is calibrated using a self-reciprocity method, and the detailed experimental process can be found in our previous work [13]. A pulse-echo immersion experiment is conducted to measure the attenuation coefficients at the frequencies of 3.5 and 7 MHz for each specimen. The wave velocity of each specimen is also determined using the time of flight method. Measurements are repeated five times, and the mean values are calculated and used. The mean attenuation coefficient and wave velocity results are shown in Table 2.

## 4. Results and Discussion

### 4.1. Microstructure Properties of the TP304 Steel Specimens

When TP304 stainless steel is located in a high temperature environment, its microstructures and mechanical properties will be changed. These changes can be evaluated by directly observing the microstructure and testing the hardness.

**Figure 3:**
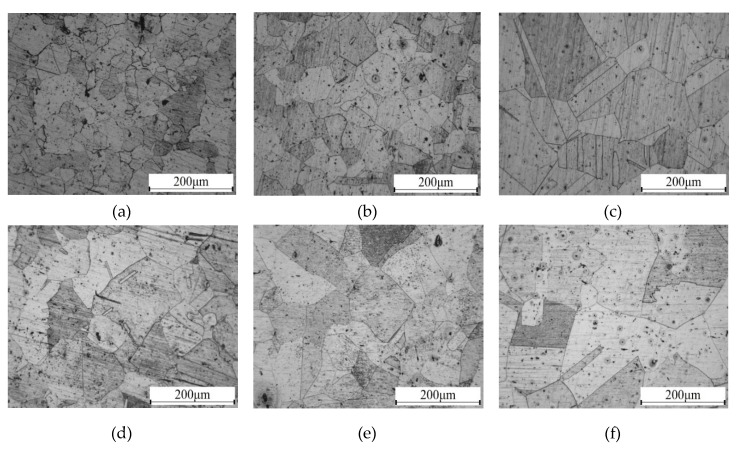
Microstructural evolution of TP304 stainless steel at six different stages: (**a**) raw material, (**b**) specimen after annealing for 2 h, (**c**) specimen after annealing for 4 h, (**d**) specimen after annealing for 6 h, (**e**) specimen after annealing for 10 h, and (**f**) specimen after annealing for 14 h.

**Figure 4:**
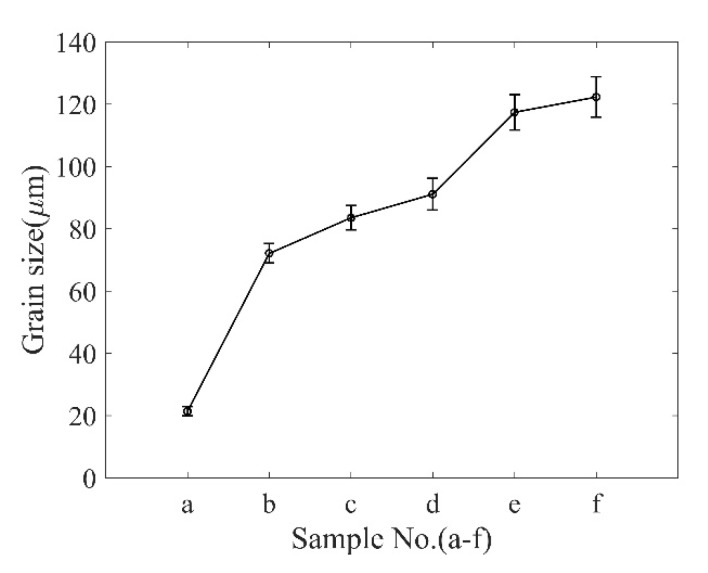
Variations in grain sizes for different specimens.

Figure 3 shows the microstructural evolution of the TP304 steel specimens at different stages. The “line intercept” method [17] was introduced to determine the grain size. Variations in grain size of the specimens at different stages are shown in Figure 4. It is observed that the grain size is about 20 μm at the initial state. New grains with bigger sizes will be generated after annealing, or the grains will grow as the annealing time increases. Thus, the grain size of the specimens is bigger than that in the specimen at the initial state, as shown in Figure 3b–f. It is also found that precipitated phases, such as chromium, appear after a long annealing time.

**Figure 5:**
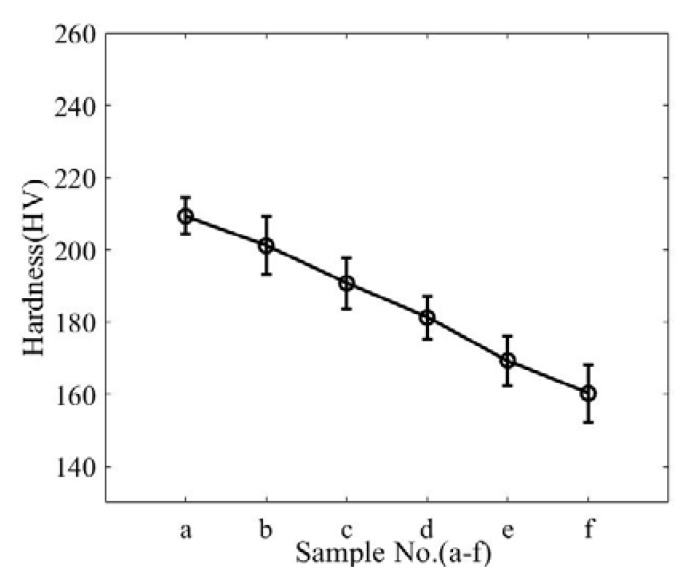
Variations in hardness for different specimens.

The increase in the grain size will reduce the mechanical properties of the TP304 steel. One of the performance degradation behaviors is that the hardness decreases. The hardness of each specimen was tested by a Vickers hardness tester at 5 kg load and 10 s dwell time, and five indentations were made on each specimen. Figure 5 shows the average and standard deviation of the tested hardness results.

### 4.2. Nonlinear Experiment Results

**Figure 6:**
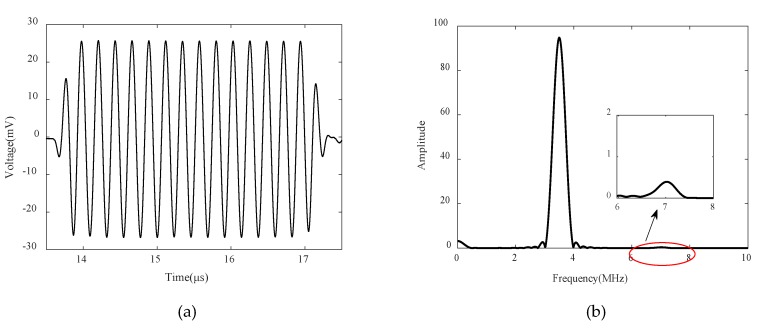
(**a**) A typical time domain signal and (**b**) its frequency properties. The signals at 3.5 and 7 MHz denote the fundamental and second harmonic wave amplitudes, respectively.

A typical ultrasonic wave signal is shown in Figure 6a. A Hanning window is employed to extract the whole toneburst signal, and fast Fourier transformation is used to analyze the frequency domain wave signal. The fundamental and second harmonic wave amplitudes in the frequency domain are shown in Figure 6b. We use a rectangular window with a width of 1 MHz to extract the fundamental and second harmonic wave signals, and calculate the corresponding wave displacement amplitudes using the transducer calibration result.

**Figure 7:**
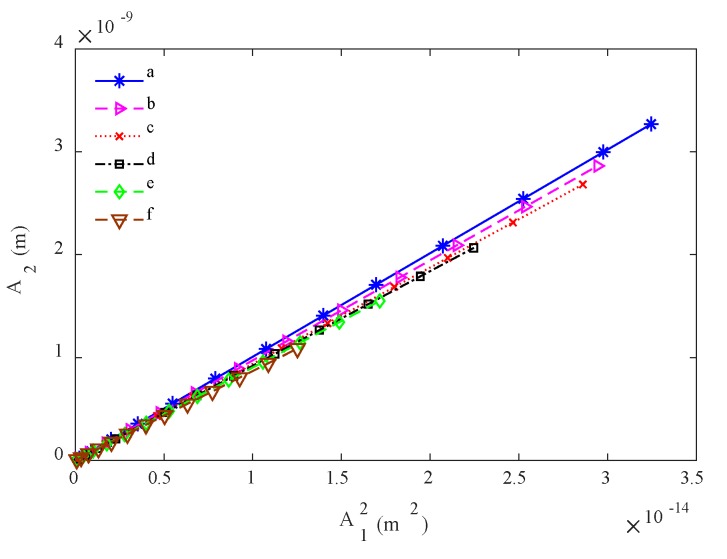
The slopes of A_2_/A_1_^2^ for different specimens.

The results of ${\tilde{A}}_{2}/{\tilde{A}}_{1}^{2}$ at different driving voltages from one experiment are shown in Figure 7. The nonlinear parameter can be calculated using the slope of ${\tilde{A}}_{2}/{\tilde{A}}_{1}^{2}$, as Equation (1) indicates. The initial nonlinear parameter of the TP304 steel determined without any heat treatment is 3.1. The corrections for diffraction, attenuation and reflection coefficient can be calculated using Equations (3) and (4) with the measured attenuation coefficients and wave velocity, and when all these corrections are made, the nonlinear parameter becomes 11.4. In order to verify the validity of the present pulse-echo nonlinear method, the nonlinear parameter of this TP304 steel specimen is also measured using the traditional through-transmission method [13]. When the effects of diffraction and attenuation are taken into account, the nonlinear parameter of this specimen is measured as 12.2. The agreement of the nonlinear parameters determined using different methods demonstrates that the proposed pulse-echo nonlinear experiment is effective. It should be stated that when the through-transmission method is used, the accuracy will be affected by the misalignment and the clamping state for the two transducers. Our repeated experiments show that the differences in nonlinear parameters determined using the pulse-echo method are smaller than those determined using the through-transmission method. Thus, the nonlinear parameters determined will be more stable with a higher accuracy.

The same method is used to determine the absolute nonlinear parameters of specimens with different microstructural evolution. The mean results before any corrections are shown in Figure 7, and the determined nonlinear parameter values are displayed in Table 3. One can find, from the initial determined results, that the nonlinear parameters decrease as the annealing time increases. However, this conclusion is inconsistent with most experiment results [18,19]. The reason is that these nonlinear parameters are affected by the diffraction, attenuation and reflection, because the attenuation coefficients and wave velocities of these specimens are different, as shown in Table 2. The measured wave velocities and attenuation coefficients are used to calculate the total corrections, and the corrected results are shown in Table 3. It is found that the results before and after corrections show different variation tendencies. Thus, the use of initial results will lead to drawing erroneous conclusions. It should also be stressed that although there are errors for these measurement results, the variation tendency of the results for different specimens does not change.

### 4.3. Relationship Between the Microstructural Evolution and Nonlinear Parameters

**Figure 8:**
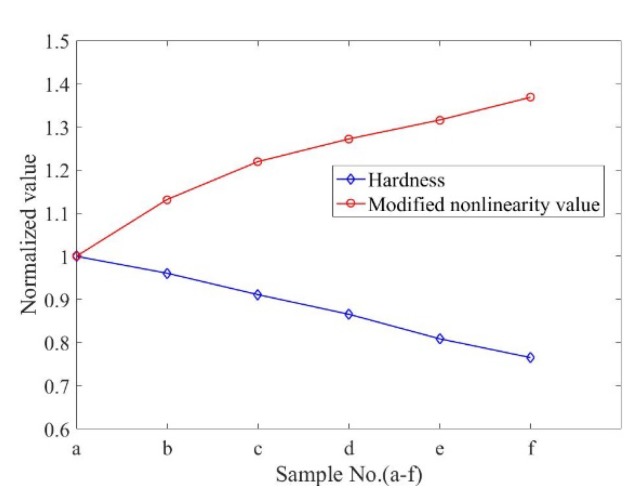
Variations in nonlinear parameters in steel specimens with different microstructures.

Since the heat treatment is conducted in laboratory conditions and due to the limited number of specimens, it is unscientific to establish the relationship between the nonlinear parameter and the performance degradation of the TP 304 stainless steel in its whole life cycle [20]. However, we can still obtain some basic findings from these experimental results. It is shown that, as the annealing time increases, the microstructure of the TP304 stainless steel will evolves in different degrees. The microstructural evolution can be reflected by the changes of mechanical properties of TP304 steels, and also can be evaluated by measuring nonlinear parameters. Figure 8 shows the variations in the nonlinear parameter and hardness for the TP304 stainless steel specimens with different microstructures. First, as the annealing time increases, the grain sizes increase continuously and the hardness decreases. As the grain sizes increase, the interatomic binding force and the modulus of elasticity decrease, which causes the wave velocity to decrease. The scattering is enhanced due to large grains, so the attenuation coefficients increase as the annealing time increases [21]. The grain boundary is reduced when the grain size increases, and the chromium element is precipitated, which will increase the nonlinear parameter. In addition, microcrack is more likely to occur when the grain size increases, and microcrack is one of the important factors that tends to increase the nonlinear parameter [22,23].

There is a significant increase in the nonlinear parameters in the specimens after the annealing process. This interesting finding shows that the use of the nonlinear parameters can be effective in characterizing the material degradations. However, there will be different tendencies of nonlinear parameters with the property degradation for different materials [12,18,19,24]. The lack of consideration of attenuation effects may be one reason for this. In this work, it is observed that the measured nonlinear parameters are different before and after the attenuation corrections are made. Therefore, for the materials with strong attenuation, the effects of attenuation must be considered when the nonlinear parameter is measured.

It is also found that the attenuation coefficients and wave velocities change as the material’s microstructures vary. The variations in attenuation coefficients and wave velocities are mainly related to the change in grain sizes. However, in certain cases, the degradation of the mechanical properties is induced by the micro-flaws or fatigue damage, and the traditional ultrasonic wave parameters are insensitive to these changes [24,25,26]. The nonlinear acoustics will have more obvious advantages in characterizing the material’s mechanical properties.

When nonlinear acoustics are used, for accurately characterizing material properties, corrections for the diffraction and attenuation are essential to determine the nonlinear parameter. In addition, rather than using the relative nonlinear parameter for evaluation, the absolute nonlinear parameter has the advantage of quantitatively characterizing the material properties [9]. The proposed nonlinear pulse-echo method can be used to determine the absolute nonlinear parameters; thus, it can benefit the application of nonlinear acoustics for material characterization in practical tests.

## 5. Conclusions

The microstructural evolution of the TP304 stainless steel is characterized using a pulse-echo nonlinear method. The impendence mismatch problem in the experimental setup is solved and a correction theory is presented to determine the absolute nonlinear parameter. The measured nonlinear parameters of the TP304 stainless steel specimen using the present method and the through-transmission method agree with each other, which reveals that the present method is effective. The pulse-echo method enables single-side access to the test component, and provides a useful tool for the practical application of nonlinear ultrasonic measurement. Measurement of the absolute nonlinear parameter can be achieved using the pulse-echo method, a technique that is helpful for quantitatively evaluating material properties.

The nonlinear parameters of annealed TP304 steel specimens are measured. It is shown from these measurement results that as the annealing time increases, the mechanical properties are degraded, which is embodied in the larger grain size and reduced hardness. The evolution of their microstructure is reflected by the variations in the acoustic nonlinear parameter, as the performance degradation of the TP304 stainless steel will enhance the nonlinear harmonic generation and increase the nonlinear parameters.

## Figures and Tables

**Table 1:** Chemical composition of TP304 stainless steel (wt%).

C	Si	Mn	P	S	Cr	Ni
0.035	0.385	1.270	0.052	0.014	17.10	8.01

**Table 2:** Wave velocity and attenuation results in different specimens.

Specimen	α_1_ (Np/m)	α_2_ (Np/m)	*v* (m/s)
(a)	4.5	18.0	5728.4
(b)	6.9	27.1	5725.7
(c)	7.6	28.9	5718.4
(d)	8.3	31.7	5685.9
(e)	9.0	34.8	5657.7
(f)	9.9	37.9	5583.7

**Table 3:** Nonlinear parameters for different specimens before and after corrections.

Specimen	a	b	c	d	e	f
*β* (Before correction)	3.1	2.9	2.7	2.6	2.6	2.5
*β* (After correction)	11.4	12.9	13.9	14.5	15.0	15.6
